# Fibrovascular pigment epithelial detachment in eyes with subretinal hemorrhage secondary to neovascular AMD or PCV: a morphologic predictor associated with poor treatment outcomes

**DOI:** 10.1038/s41598-020-72030-6

**Published:** 2020-09-10

**Authors:** Jae Hui Kim, Joo Yeon Kim, Dong Won Lee, Chul Gu Kim, Jong Woo Kim

**Affiliations:** Department of Ophthalmology, Kim’s Eye Hospital, #156 Youngdeungpo-dong 4gaYoungdeungpo-gu, Seoul, 150-034 South Korea

**Keywords:** Macular degeneration, Outcomes research, Prognostic markers

## Abstract

To evaluate the influence of fibrovascular pigment epithelial detachment (FVPED) on treatment outcomes in eyes with subretinal hemorrhage secondary to neovascular age-related macular degeneration (AMD) and polypoidal choroidal vasculopathy (PCV). This retrospective study included 83 eyes diagnosed with fovea-involving submacular hemorrhage secondary to neovascular AMD or PCV. All the patients were treated with intravitreal anti-vascular endothelial growth factor. Eyes showing definite FVPED, which involves the subfoveal region, were included in the FVPED group. Eyes without subfoveal PED, shallow irregular PEDs, or serous/hemorrhagic PED were stratified to the non-FVPED group. The best-corrected visual acuity (BCVA) at diagnosis, at 3 months, at 12 months, and lesion re-activation after initial treatment were compared between the two groups. The mean size of hemorrhage was 8.6 ± 7.6 disc diameter areas. In the FVPED group, the mean logarithm of minimal angle of resolution BCVA was 1.11 ± 0.49 at diagnosis, 0.89 ± 0.58 at 3 months, and 1.05 ± 0.63 at 12 months. In the non-FVPED group, the values were 0.97 ± 0.56, 0.56 ± 0.55, and 0.45 ± 0.50, respectively. The BCVA at 3 months (*P* = 0.036) and at 12 months (*P* < 0.001) was significantly worse in the FVPED group than in the non-FVPED group. In addition, the incidence of lesion reactivation was greater in the FVPED group (83.3%) than in the non-FVPED group (38.5%) (*P* < 0.001). The presence of subfoveal FVPED was associated with a high incidence of lesion re-activation and poor treatment outcomes in eyes with subretinal hemorrhage. This result suggests that different treatment strategies are needed between eyes with and without FVPED.

## Introduction

Age-related macular degeneration (AMD) is one of the main etiologies of vision impairment^1^. Considering the increase in its global prevalence^2^. along with an increase in the population of patients who underwent long-term active treatment^3^, AMD may become an even more important disorder in the future. Subretinal hemorrhage is a frequently noted finding in neovascular age-related macular degeneration (AMD) and polypoidal choroidal vasculopathy (PCV)^4^. Subretinal hemorrhage is considered to be associated with poor prognosis in neovascular AMD^5,6^. In animal experiments, diffuse degeneration of retinal tissue was noted after subretinal hemorrhage^7,8^. In OCT studies, marked degeneration and thinning of the outer retinal layers were noted after resolution of subretinal hemorrhage^9,10^.

Because of the negative impact of subretinal hemorrhage, previous studies have mainly focused on the ‘hemorrhage’ itself and attempted to address questions about how to remove effectively the hemorrhage from the fovea^11,12^ or whether the amount or location of the hemorrhage affects the treatment outcome^13–16^. Although, various morphologic predictors have been reported in neovascular AMD^17,18^, the influence of other factors in hemorrhage cases has not been fully elucidated yet.

Pigment epithelial detachment (PED) is one of the morphologic predictors of treatment outcomes in neovascular AMD^17,18^. In particular, fibrovascular PED (FVPED) has been reported to be associated with non-response to treatment^19^ and long-term visual deterioration^20^. Despite these previous observations, the impact of PED on treatment outcomes has not been specifically evaluated yet in subretinal hemorrhage cases.

Anti-vascular endothelial growth factor (VEGF) therapy is a useful treatment option for treating submacular hemorrhage secondary to neovascular AMD or PCV. Marked improvement in visual acuity accompanied with anatomic stabilization was noted after the therapy^13,21,22^. The purpose of the present study was to evaluate the impact of FVPED on treatment outcomes in eyes with subretinal hemorrhage secondary to neovascular AMD and PCV.

## Materials and methods

This retrospective, observational study was performed at a single center. The study was approved by the Institutional Review Board of Kim’s Eye Hospital and was conducted in accordance with the tenets of the Declaration of Helsinki. Due to the retrospective nature of this study, the need for an informed consent was waived off (Kim’s Eye Hospital IRB, Seoul, South Korea).

### Patients

The present study was performed in patients who were diagnosed with subretinal hemorrhage secondary to neovascular AMD and PCV between January 2017 and June 2018. Only fovea-involving hemorrhages greater than 1 disc diameter in size were included.

The exclusion criteria were as follows: (1) less than 12 months of follow-up after diagnosis, (2) myopia of -6.0 D or greater and an axial length of 26.0 mm or greater, (3) concomitant retinal vascular disorders (e.g., macroaneurysms, proliferative diabetic retinopathy, and retinal vascular occlusion), (4) severe media opacity, which may preclude accurate image acquisition. Eyes that received vitrectomy due to development of dense vitreous hemorrhage were not excluded from the study. However, eyes with dense vitreous hemorrhage that did not receive vitrectomy were excluded from the study because vitreous hemorrhage could cause significant bias when evaluating the visual outcome. Thickness of the subretinal hemorrhage was not included in the eligibility criteria. If both eyes satisfied eligibility criteria, only the eye with earlier symptoms was included in the study.

### Examinations

Ophthalmological examination, including the measurement of best-corrected visual acuity (BCVA) and a 90-D-lens slit-lamp biomicroscopy evaluation, was performed. Fundus imaging results were obtained using CX-1(Topcon, Tokyo, Japan). Fluorescein angiography and indocyanine-green angiography (ICGA) images were obtained using Spectralis HRA + OCT(Heidelberg Engineering, Heidelberg, Germany). During diagnosis, optical coherence tomography (OCT) images were acquired using the Spectralis HRA + OCT device. During the follow-up, a Spectralis HRA + OCT or an RS 3,000 (Nidek Co., Ltd., Tokyo, Japan) device was used to acquire OCT images.

Classification of neovascular AMD and PCV was performed based on indocyanine green angiography (ICGA) results. PCV was diagnosed by the presence of polypoidal lesions with or without branching vascular networks^23,24^. Cases without PCV features were classified as neovascular AMD. Two independent examiners (J.H.K. and S.H.L.) analyzed the ICGA results. Any discrepancies were settled by discussion between the 2 examiners. Eyes without an ICGA result or an accurate classification were defined as unclassified.

Central retinal thickness (CRT) was defined as the average thickness of the central macular 1-mm area, which was automatically calculated by the OCT software. The size of subretinal hemorrhage was measured based on fundus photographs using Image J (National Institute of Health, Bethesda, MD, USA).

### Treatment and follow-up

The treatment and follow-up methods used in this study were similar to those in our previous study^25^. Patients were initially administered 3 monthly injections. Ranibizumab (0.5 mg/0.05 mL of LUCENTIS; Genentech Inc., San Francisco, CA, USA) or aflibercept (2.0 mg/0.05 mL of EYLEA; Regeneron, Tarrytown, NY, USA) was used for the initial treatments. After an initial treatment, re-treatment was performed on an as-needed basis. One of 3 anti-VEGF agents—ranibizumab, aflibercept, or bevacizumab (1.25 mg/0.05 mL of AVASTIN; Genentech Inc.)—was used for the additional treatment. If the treating physician determined that a more effective treatment was required to preserve vision, the treatment regimen was changed from the as-needed regimen to the proactive regimen. In some patients, treatment was discontinued at the physician’s discretion.

### Comparisons between the FVPED group and the non-FVPED group

**Figure 1:**
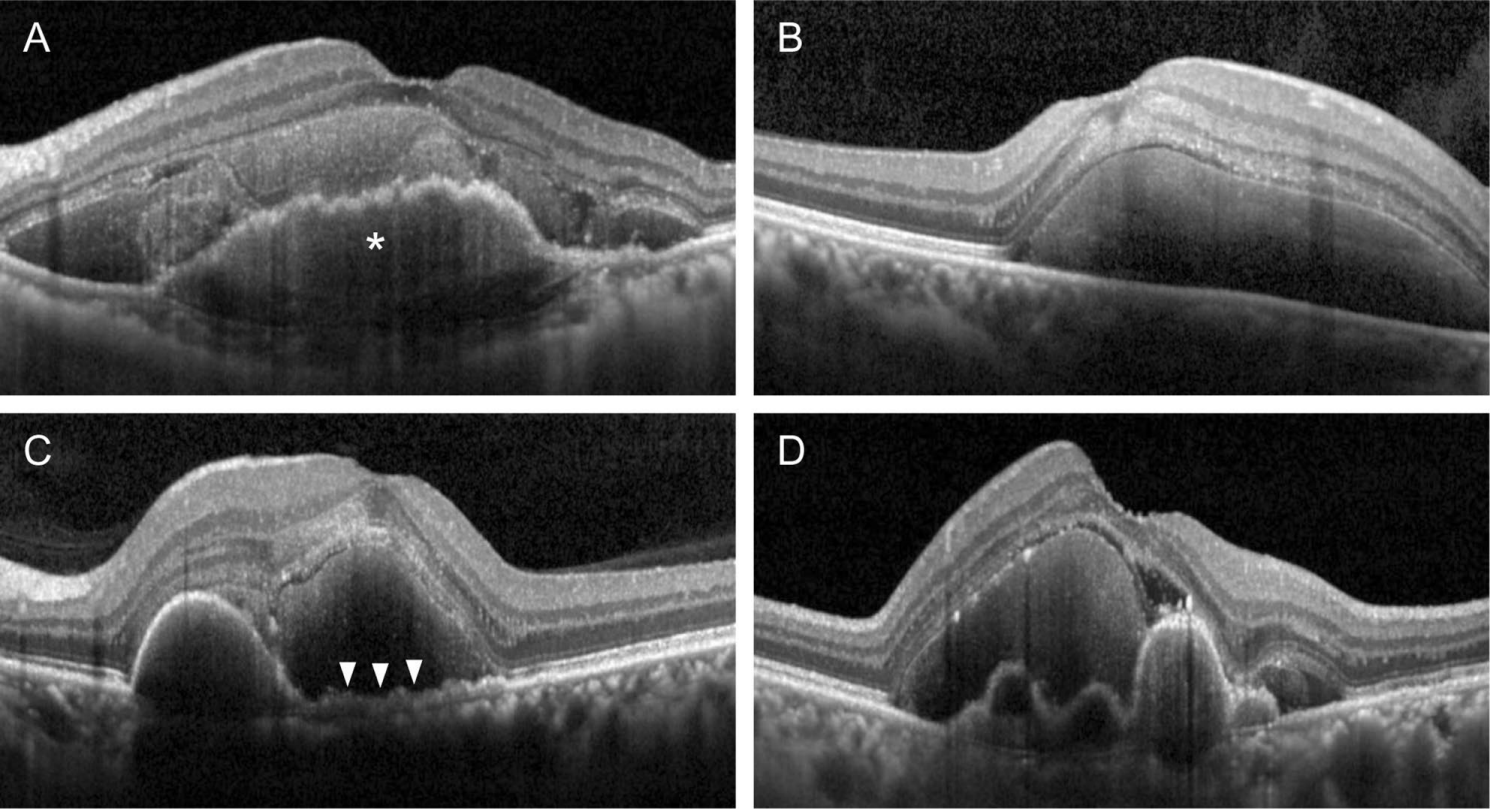
Optical coherence tomography images of eyes with subretinal hemorrhage showing classification of included eyes based on the characteristic of pigment epithelial detachment (PED). Eyes showing subfoveal fibrovascular PED (**A**, asterisk) were classified as the fibrovascular PED group. Eyes showing no subfoveal PED (**B**), shallow irregular PEDs (**C**, arrowheads), or serous/hemorrhagic PED (**D**) were classified as the non-fibrovascular PED group.

Based on the OCT finding at diagnosis, included eyes were divided into 2 groups: the FVPED and the non-FVPED group. Eyes showing definite FVPED, which involves the subfoveal region (Fig. 1A), were included in the FVPED group. Eyes showing no subfoveal PED (Fig. 1B), shallow irregular PEDs (Fig. 1C), or serous/hemorrhagic PED (Fig. 1D) were classified as the non-FVPED group. Eyes with FVPED, without involvement of the subfoveal region, were also included in the non-FVPED group. Angiography images were not used to diagnose FVPED.

The classification of the 2 groups was performed by 2 independent examiners (J.H.K. and S.H.L.). Any discrepancies were settled by the discussion between the 2 examiners. It is reported that serous PED can sometimes mask the existence of FVPED^20^. Thus, OCT images taken at 3 months were additionally analyzed when thick subretinal hemorrhage precluded accurate classification of the underlying PED.

The following baseline characteristics were compared between the 2 groups; age, sex, diabetes mellitus, hypertension, use of anticoagulants, type of neovascularization (neovascular AMD vs PCV), size of hemorrhage, CRT, thickness of the subfoveal hemorrhage, and type of anti-VEGF drug for loading injections. In addition, the type of neovascularization was determined for eyes in which accurate classification of neovascular AMD and PCV could be performed. The BCVA at diagnosis, at 3 months, and at 12 months was compared between the 2 groups. In addition, the incidence of fibrotic scar and tear in the retinal pigment epithelium (RPE) at 12 months were estimated. If the patient failed to visit exactly at 3 or 12 months, values from the other visits, which were closest to the 3 or 12 months, were used for the analysis. The thickness of the subfoveal hemorrhage was defined as the vertical distance between the photoreceptor and RPE layers at the foveal lesion.

The number of anti-VEGF injections during the 12 months was compared between the 2 groups. In addition, the difference in the lesion re-activation during the first 12 months was also compared. In the lesion re-activation analysis, only eyes, in which complete resolution of fluid without increase in or new development of subretinal hemorrhage was noted after 3 initial loading injections were included.

### Statistical analyses

Statistical analyses were performed using a commercially available software package (SPSS ver. 12.0 for Windows; SPSS Inc., Chicago, IL, USA). Differences in baseline characteristics between the FVPED group and the non-FVPED group were analyzed using independent samples t-test, chi-square test, or Fisher’s exact test. Difference in lesion re-activation between the 2 groups was compared using a Kaplan–Meier survival analysis with log rank test. Difference in the number of anti-VEGF injections administered between the 2 groups was compared using an independent samples t-test. BCVAs were compared using independent samples t-test with Bonferroni’s correction. Mean values were presented as mean ± standard deviation (SD). For all tests, a *P* value < 0.05 was considered significant.

## Results

A total of 84 eyes satisfied eligibility criteria. Among them, one eye was not included in the result analysis. In this eye, dense vitreous hemorrhage developed during the loading phase but vitrectomy to clear the vitreous hemorrhage was not performed. Ultimately, 83 eyes (83 patients) were included in the study. The mean age was 69.2 ± 9.1 years. Baseline characteristics of the patients are summarized in Table 1.

**Table 1:** Baseline characteristics of the study population (n = 83).

Characteristics	Values
Age, years	69.2 ± 9.1
Sex, male:female	61 (73.5%):22 (26.5%)
Diabetes mellitus	24 (28.9%)
Hypertension	52 (62.7%)
Use of anticoagulants	18 (21.7%)
Presence of subfoveal fibrovascular PED	28 (33.7%)
**Type of neovascularization**	
Neovascular AMD	27 (32.5%)
PCV	49 (59.0%)
Undetermined	7 (8.4%)
Size of hemorrhage, disc diameter areas	8.6 ± 7.6
Central retinal thickness (μm)	581.7 ± 213.7
Subfoveal hemorrhage thickness (μm)	291.5 ± 192.5

The data are presented as mean ± standard deviation or No. (%) where applicable.

*PED* pigment epithelial detachment, *AMD* age-related macular degeneration, *PCV* polypoidal choroidal vasculopathy, *logMAR* logarithm of minimal angle of resolution.

Twenty-seven eyes (32.5%) were classified as neovascular AMD and 49 eyes (59.0%) were classified as PCV. The remaining 7 eyes (8.4%) were unclassified. Aflibercept was used for the initial loading injections in 74 eyes (89.2%), whereas ranibizumab was used in the remaining 9 eyes (10.8%). During the 12-month follow-up period, the following anti-VEGF drugs were used: aflibercept only = 70 eyes (84.3%), aflibercept and bevacizumab = 4 eyes (4.8%), ranibizumab only = 5 eyes (6.0%), ranibizumab and bevacizumab = 2 eyes (2.4%), and ranibizumab and aflibercept = 2 eyes (2.4%).

Twenty-eight eyes (33.7%) were included in the FVPED group and 55 eyes (66.3%) were included in the non-FVPED group (Table 2).

**Table 2:** Comparisons of baseline characteristics between the fibrovascular pigment epithelial detachment (FVPED) group and the non-FVPED group.

Characteristics	FVPED group (n = 28)	Non-FVPED group (n = 55)	*P* value
Age, years	73.5 ± 7.9	67.1 ± 8.9	0.001^†^
Sex, male:female	17 (60.7%):11 (39.3%)	44 (80.0%):11 (20.0%)	0.071^‡^
Diabetes mellitus	9 (32.1%)	15 (27.3%)	0.644^‡^
Hypertension	21 (75.0%)	31 (56.4%)	0.097^‡^
Use of anticoagulants	7 (25.0%)	11 (20.0%)	0.601^‡^
Type of neovascularization*			< 0.001^‡^
Neovascular AMD	17 (70.8%)	10 (19.2%)	
PCV	7 (29.2%)	42 (80.8%)	
Size of hemorrhage, disc diameter areas	5.9 ± 6.3	9.9 ± 7.9	0.013^†^
Central retinal thickness (μm)	551.1 ± 223.8	597.3 ± 208.7	0.368^†^
Subfoveal hemorrhage thickness (μm)	252.9 ± 219.3	311.1 ± 176.3	0.330^†^
**Type of anti-VEGF drug for loading injections**			0.157**
Aflibercept	23 (82.1%)	51 (92.7%)	
Ranibizumab	5 (17.9%)	4 (7.3%)	

The data are presented as mean ± standard deviation or No. (%) where applicable.

*AMD* age-related macular degeneration, *PCV* polypoidal choroidal vasculopathy, *logMAR* logarithm of minimal angle of resolution.

*Statistical analysis was performed for 24 eyes with the fibrovascular PED group and 52 eyes in the non-fibrovascular PED group in which accurate classification of neovascular AMD and PCV can be performed.

^†^Statistical analysis with independent samples *t* test.

^‡^Statistical analysis with chi-square tests.

**Statistical analysis with Fisher’s exact test.

The classification of 79 eyes (95.2%) to FVPED or non-FVPED groups was based on the OCT images acquired at diagnosis. In the remaining 4 eyes (4.8%), this classification was based on the OCT images acquired at 3-month follow-up. Among the 4 eyes, one was included in the FVPED group and 3 in the non-FVPED group.

The FVPED group was significantly older than the non-FVPED group (mean 73.5 ± 7.9 years old vs mean 67.1 ± 8.9 years old, respectively) (*P* = 0.001), and the size of hemorrhage was significantly smaller in the FVPED group than in the non-FVPED group (mean 5.9 ± 6.3 disc diameter areas vs mean 9.9 ± 7.9 disc diameter areas) (*P* = 0.013). In addition, the proportion of neovascular AMD was significantly higher in the FVPED group than in the non-FVPED group (70.8% vs 19.2%) (*P* < 0.001). Other characteristics, including sex (*P* = 0.071), diabetes mellitus (*P* = 0.644), hypertension (*P* = 0.097), use of anticoagulants (*P* = 0.097), central retinal thickness (*P* = 0.368), and thickness of the subfoveal hemorrhage (*P* = 0.330) were not different between the 2 groups.

During the 12-month follow-up period, a mean of 4.3 ± 1.3 anti-VEGF injections were administered to patients in the FVPED group, while a mean of 3.6 ± 1.0 injections were administered to those in the non-FVPED group (*P* = 0.012). Cataract surgery was performed in one eye in the FVPED group and 2 eyes in the non-FVPED group. Vitrectomy to clear the vitreous hemorrhage was performed in 2 eyes of the FVPED group and in 4 eyes in the non-FVPED group. Among the 4 eyes in the non-FVPED group, combined cataract surgery was also performed in 2 eyes. The sole purpose of vitrectomy was to remove the vitreous hemorrhage. Techniques that directly manage the subretinal hemorrhage, such as subretinal injection of tissue plasminogen activator or gas injection, were not performed.

**Figure 2:**
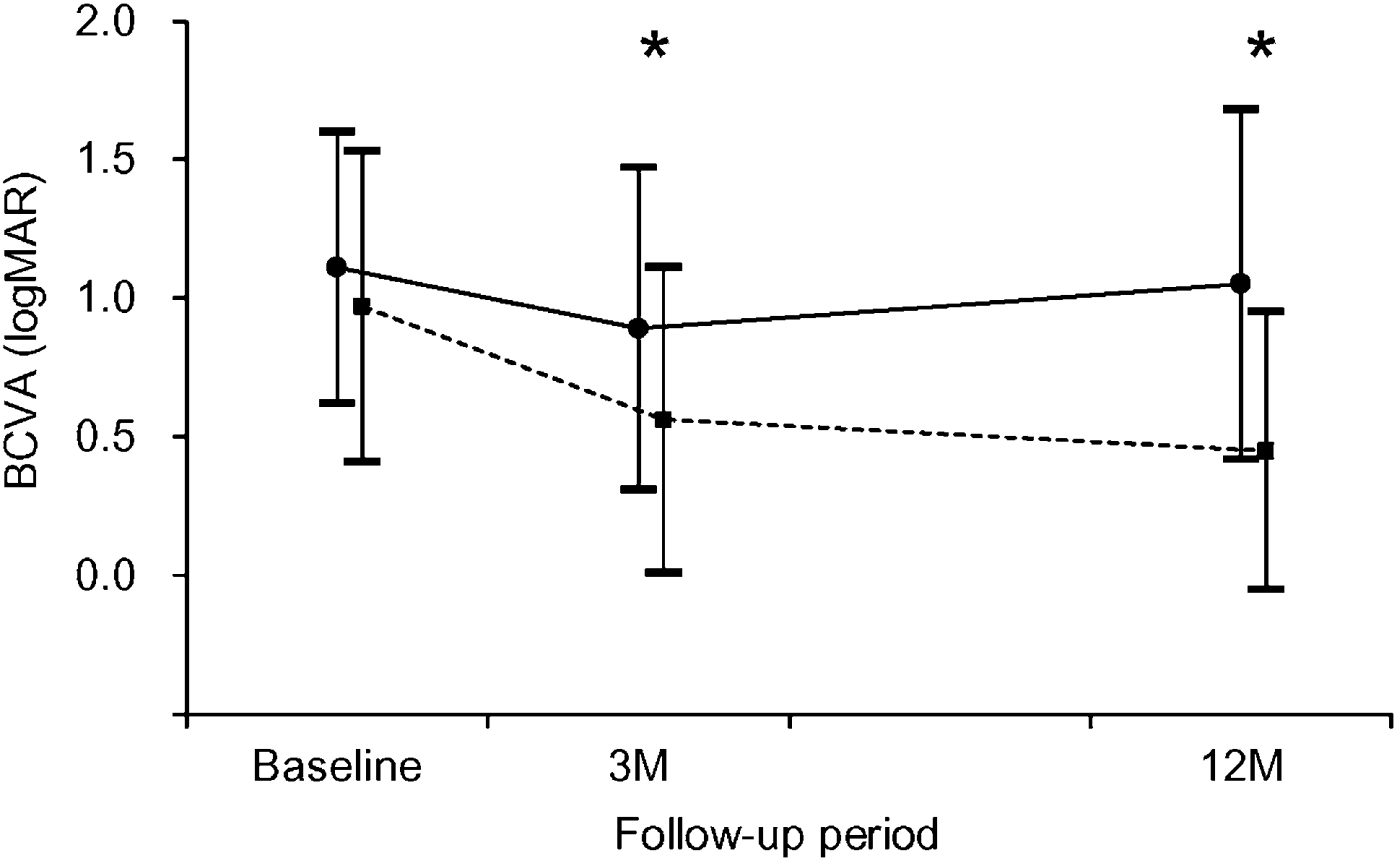
Comparison of 12-month changes in the logarithm of minimal angle of resolution (logMAR) best-corrected visual acuity (BCVA) in the fibrovascular pigment epithelial detachment (FVPED) group (solid line, N = 28) and the non-FVPED group (dotted line, N = 55). Asterisk indicates significant difference in BCVA between the 2 groups. M = month.

The mean logMAR BCVA in all 83 eyes was 1.00 ± 0.50 (Snellen equivalents = 20/200) at diagnosis, 0.67 ± 0.58 (20/93) at 3 months, and 0.65 ± 0.62 (20/89) at 12 months. In the FVPED group, the mean logMAR BCVA was 1.11 ± 0.49 (20/257) at diagnosis, 0.89 ± 0.58 (20/155) at 3 months, and 1.05 ± 0.63 (20/224) at 12 months (Fig. 2). In the non-FVPED group, the values were 0.97 ± 0.56 (20/186), 0.56 ± 0.55 (20/72), and 0.45 ± 0.50 (20/56) at diagnosis, 3 months, and 12 months, respectively (Fig. 2).

The BCVA at diagnosis was not different between the 2 groups (*P* = 0.798), whereas the BCVA at 3 months (*P* = 0.036) and at 12 months (*P* < 0.001) was significantly worse in the FVPED group than in the non-FVPED group. In the FVPED group, ≥ 2 lines of improvement in BCVA at 12 months was noted in 11 eyes (39.3%) and ≥ 2 lines of deterioration in BCVA was noted in 3 eyes (10.7%). The BCVA was maintained stable in the remaining 14 eyes (50.0%). In the non-FVPED group, ≥ 2 lines of improvement in BCVA was noted in 43 eyes (78.2%) and ≥ 2 lines of deterioration in BCVA was noted in 2 eyes (3.6%). The BCVA was maintained stable in the remaining 10 eyes (18.2%).

At 12 months, fibrotic scar was noted in 9 eyes (32.1%) and tear of RPE was noted in 3 eyes (10.7%) in the FVPED group. In the non-FVPED group, fibrotic scar was noted in 3 eyes (5.5%); none of the eyes showed tear of RPE.

**Figure 3:**
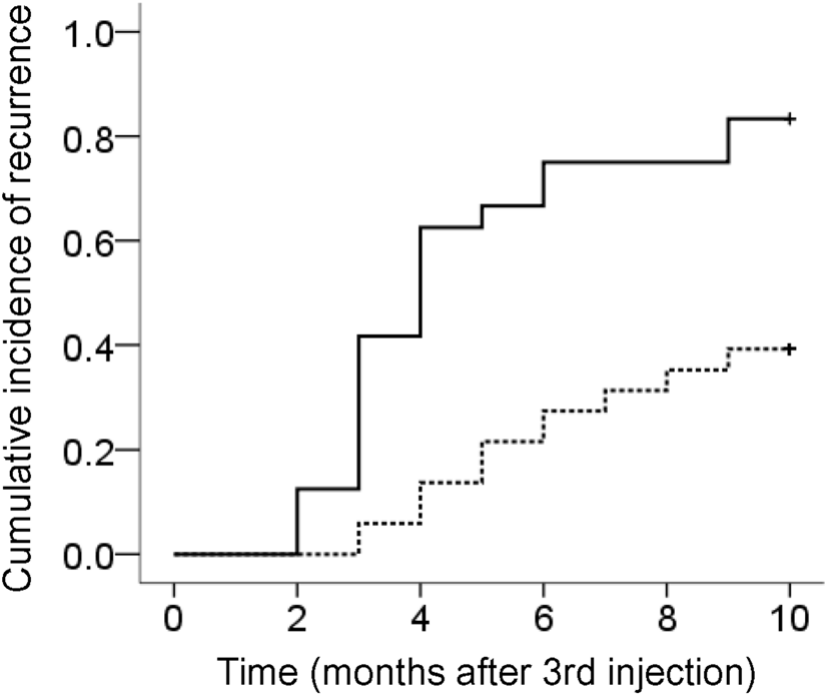
Kaplan–Meier curves showing the cumulative incidence of lesion re-activation in the fibrovascular pigment epithelial detachment (FVPED) group (solid line, N = 24) and the non-FVPED group (dotted line, N = 52).

In 76 of the 83 eyes (24 eyes in the FVPED group and 52 eyes in the non-FVPED group), complete resolution of intraretinal/subretinal fluid without increase in or new development of subretinal hemorrhage was noted after three loading injections. In the FVPED group, lesion re-activation was noted during the 12 months in 20 patients (83.3%). In the non-FVPED group, lesion re-activation was noted in 20 patients (38.5%). In Kaplan–Meier survival analysis, significant difference in the lesion re-activation was noted between the 2 groups (Fig. 3, *P* < 0.001).

Among the remaining 7 eyes without resolution of intraretinal/subretinal fluid even after 3 loading injections, the fluid resolved completely after 4–5 consecutive injections in 3 eyes (one in the FVPED group and 2 in the non-FVPED group). In these 3 eyes, re-activation was noted at mean 6.3 months (range 4–8 months) of follow-up after the last injection. In one of the 7 eyes (FVPED group), retinal pigment epithelium tear occurred during the loading injections. Therefore, additional injection was not administered. In this eye, an increase in intraretinal/subretinal fluid was noted at 5 months after the third injection. In 2 of the 7 eyes (one in the FVPED group and one in the non-FVPED group), despite remaining intraretinal/subretinal fluid, additional injections were not administered after the loading injections. An increase in the fluid was noted at 3 and 4 months after the third injection. In the remaining one eye (FVPED group), vitreous hemorrhage developed during the loading injections. Vitrectomy was performed immediately after the third injection. Postoperative examination after the vitrectomy showed persistent retinal hemorrhage and fluid. Although two additional anti-VEGF injections were administered, the intraretinal/subretinal fluid persisted.

**Figure 4:**
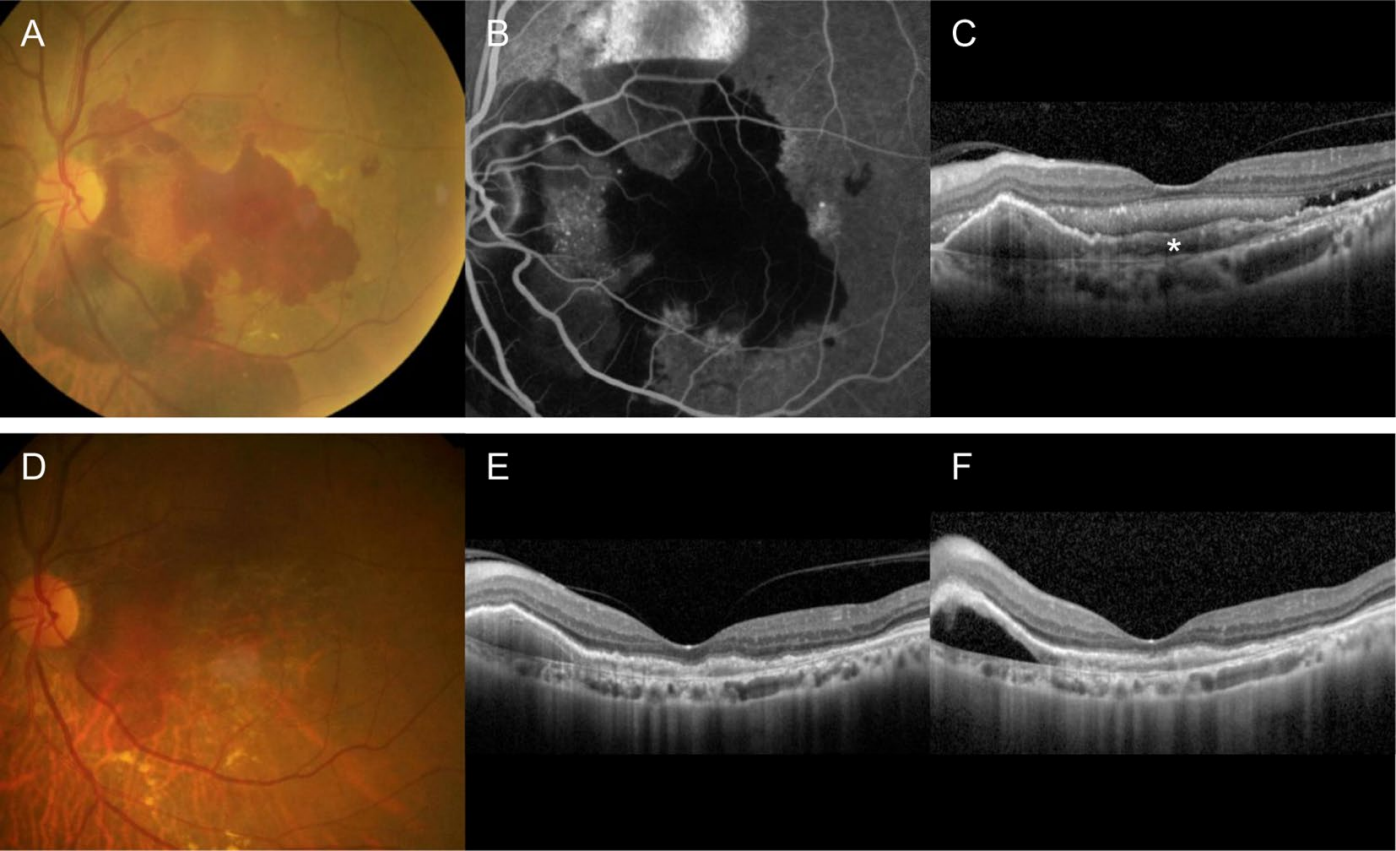
A representative case showing treatment outcome of an eye in the fibrovascular pigment epithelial detachment (FVPED) group. At diagnosis (**A**–**C**), the best-corrected visual acuity (BCVA) was 20/200 and FVPED was noted under the fovea (**C**, asterisk). One month after 3 monthly aflibercept injections, the hemorrhage was markedly resolved (**D**, **E**), and the BCVA had improved to 20/40. During the first 12 months after diagnosis, 3 aflibercept injections and 1 bevacizumab injection were additionally administered due to re-activation of the lesion. The BCVA at 12 months was 20/100 (**F**). **A**, **D** = fundus photography, **B** = fluorescein angiography, **C**, **E**, **F** = optical coherence tomography.

**Figure 5:**
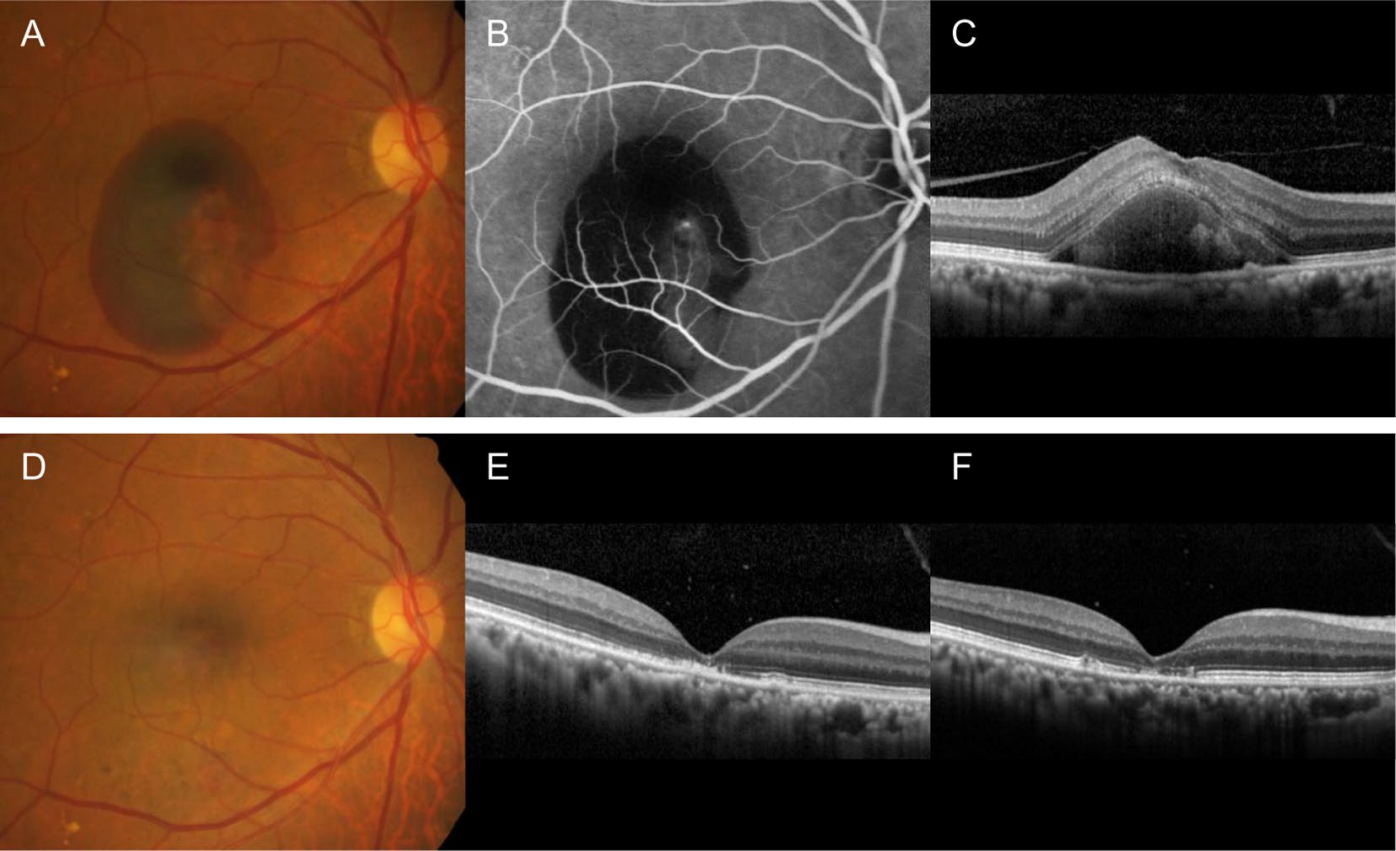
A representative case showing the treatment outcome of an eye in the non-fibrovascular pigment epithelial detachment (FVPED) group. At diagnosis (**A**–**C**), the best-corrected visual acuity (BCVA) was 20/100. One month after 3 monthly aflibercept injections, the hemorrhage was completely resolved (**D**, **E**) and the BCVA had improved to 20/50. Re-activation of the lesion was not noted during the 12-month follow-up period. The BCVA at 12 months was further improved to 20/40 (**F**). **A**, **D** = fundus photography, **B** = fluorescein angiography, **C**, **E**, **F** = optical coherence tomography.

Figures 4 and 5 show representative cases of the FVPED group and the non-FVPED group, respectively.

## Discussion

Anti-VEGF therapy is one of the widely used, effective treatment modalities for submacular hemorrhage secondary to neovascular AMD and PCV. In a previous study by Kim et al., approximately 4 lines of visual improvement were noted 6 months after treatment^13^. Similarly, a mean visual recovery of + 17 ETDRS letters was noted at 12 months in the study by Shienbaum et al.^22^ In the study by Altaweel et al., eyes with > 50% of the lesion composed of hemorrhage showed similar treatment outcomes when compared to eyes with less or no blood^21^. In the present study, overall visual improvement after treatment was comparable to that observed in previous studies^13,22^.

FVPED has been reported to be closely associated with poor visual prognosis in neovascular AMD. Suzuki et al., demonstrated that FVPED is associated with non-response to ranibizumab therapy^19^. In a study by Hoerster, the volume of FVPED was correlated more significantly with poor 24-month visual acuity than with other factors, such as subretinal fluid, subretinal tissue, and serous PED^20^. In addition, FVPED is also a risk factor of RPE tears^26^. In the present study, there was a marked difference in the treatment outcome between eyes with and without fovea-involving FVPED. Higher incidence of re-activation was noted and higher number of anti-VEGF injections was required in the FVPED group than in the non-FVPED group. In addition, the visual outcome was markedly worse in the FVPED group than in the non-FVPED group.

It is well established that lesion re-activation is not noted in some eyes after initial anti-VEGF treatment^27,28^. In PCV, subretinal hemorrhage is associated with a low risk of lesion reactivation^29^. In the study by Kim et al., lesion re-activation after initial treatment during the first year was noted in 47.8% of the eyes with subretinal hemorrhage^29^. In the study by Baek et al., injection frequency has significantly decreased after the development of subretinal hemorrhage^30^. In the present study, the overall incidence of lesion re-activation was slightly higher than in a previous PCV study^29^. However, there was a marked difference in the incidence between the FVPED group and the non-FVPED group. In particular, the incidence in the FVPED group was even slightly higher than that reported in ordinary neovascular AMD and PCV^27,28^. It is possible that this difference in lesion re-activation might primarily contribute to the significant difference in the number of anti-VEGF injections administered between the 2 groups.

We believe the difference in lesion re-activation is an important finding because it suggests the need for different treatment strategies, influenced by the presence of FVPED. Proactive regimens, such as treat-and-extend and fixed dosing regimen, are widely used, effective treatment regimens for neovascular AMD and PCV^31–34^. However, according to Kuroda et al.^27^, one of the major drawbacks of proactive regimens is that unnecessary injections may be administered even in cases without re-activation. An as-needed regimen is another commonly used treatment method^35,36^. The efficacy of as-needed regimen has been reported to be slightly inferior than that of the proactive regimen^37^. However, since additional injections are administered only when the lesion re-activation is noted, redundant injections can be avoided in cases without re-activation. We suggest that as-needed regimen may have some benefit in subretinal hemorrhage cases without FVPED. Conversely, eyes with FVPED can be a good candidate for proactive regimen. Of course, the treatment strategy should be determined on a case-by-case basis after full discussion with the patient.

The reason why the FVPED group showed worse visual outcome than the non-FVPED group is not clear. We postulate the potential reasons as follows. Firstly, higher incidence of lesion re-activation may have a negative influence on the visual outcome. In a previous PCV study, authors postulated that the hemorrhage might have originated from the rupture of a relatively large, active, vascular lesion, and it might reduce the risk of re-activation^29^. However, neovascular tissue within the FVPED may not resolve, even though part of the tissue ruptures or bleeds. Thus, development of hemorrhage may not influence the activity of the lesion in FVPED cases.

Secondly, the presence of FVPED at the time of hemorrhage development may suggest that choroidal neovascularization has been present a relatively long time before the hemorrhage. In the present study, the central retinal thickness and the size of hemorrhage was relatively greater in the non-FVPED group than in the FVPED group. Nevertheless, the BCVA at diagnosis was relatively worse in the FVPED group than in the non-FVPED group. It is possible that the long-standing choroidal neovascularization may have damaged the retinal tissue to a certain degree, and therefore, the restoration of visual acuity was limited even after the treatment. It is also possible that the overlying RPE and photoreceptors were damaged by FVPED itself. In the study by Suzuki et al. initial FVPED was associated with non-response to ranibizumab therapy^19^. The authors suggested that FVPED decreased RPE and photoreceptor functions and was eventually responsible for the non-response^19^. In the present study, the RPE and photoreceptor that were already compromised because of FVPED may have influenced the worse visual outcome in the FVPED group. In contrast, in the non-FVPED group, the RPE and photoreceptor may be relatively healthy and might recover well with subretinal hemorrhage resolution. However, proving these hypotheses is beyond the scope of the present study. Regardless of the reason, however, the poor treatment outcomes in FVPED group suggest the need for close monitoring and aggressive treatment for these eyes. In addition, it would be necessary to inform the patients that the treatment outcome may not be satisfactory.

In the present study, both neovascular AMD and PCV cases were analyzed together. There has been controversy as to whether PCV is a subtype of neovascular AMD. Recently, Li et al. demonstrated, using histopathologic evidence, that PCV is a subtype of type 1 neovascularization rather than a distinct entity^38^. Thus, we postulate that including PCV cases may not significantly bias the result. However, considering the different nature of the two disorders, further studies with larger study populations are required to more accurately show the outcome within each diagnosis group.

This study was the first to focus on the clinical importance of FVPED in eyes with subretinal hemorrhage but has certain limitations. First, it was a retrospective study with a relatively small sample size. Second, the included patients were treated in a clinical setting. Therefore, a strict monthly follow-up was not performed, and variable follow-up intervals were set at the discretion of the clinician. Thus, the timing of re-activation could not be accurately estimated in some patients. In addition, some of our patients may have been undertreated. Third, all the patients underwent anti-VEGF monotherapy as an initial treatment. A combination of pneumatic displacement and anti-VEGF therapy may achieve superior treatment outcome than anti-VEGF monotherapy^16^. Thus, our results may not be valid when using other treatment modalities, such as pneumatic displacement with or without tissue plasminogen activator injection. In addition, when performing vitrectomy, techniques that directly manage the subretinal hemorrhage were not performed. Forth, although aflibercept was used in the majority of the included eyes, the impact of using three different anti-VEGF drugs on the study results cannot be completely neglected. Fifth, only OCT images were used to identify subfoveal FVPED. In general, both angiography and OCT can diagnose FVPED^39^. In the present study, however, angiography was not used because hemorrhage often impeded the accurate identification of leakage from the FVPED at the foveal lesion. Nevertheless, diagnosing FVPED using only OCT is a study limitation. Sixth, the stratification of 4.8% of the included eyes to FVPED and non-FVPED groups was performed based on OCT images acquired at 3-month follow-up. Since the size of FVPED can change after anti-VEGF injections^40^, it is possible that the presence of FVPED was not accurately identified in some eyes. Lastly, classification of the FVPED group and non-FVPED group was based on a qualitative method.

In summary, we investigated whether the presence of subfoveal FVPED influences the treatment outcomes in eyes with submacular hemorrhage secondary to neovascular AMD and PCV. As a result, eyes with FVPED showed higher incidence of re-activation and poor 12-month treatment outcome. This result suggests that different treatment strategies are needed between eyes with and without FVPED.

## Data Availability

The datasets generated during and/or analysed during the current study are available from the corresponding author upon reasonable request.
